# Effects of Knowing the Task’s Duration on Soccer Players’ Positioning and Pacing Behaviour during Small-Sided Games

**DOI:** 10.3390/ijerph17113843

**Published:** 2020-05-28

**Authors:** Ricardo Ferraz, Bruno Gonçalves, Diogo Coutinho, Rafael Oliveira, Bruno Travassos, Jaime Sampaio, Mário C. Marques

**Affiliations:** 1 Department of Sport Sciences, University of Beira Interior, 6200-001 Covilhã, Portugal; bfrt@ubi.pt (B.T.); mariomarques@mariomarques.com (M.C.M.); 2 Research Centre in Sports, Health and Human Development, CIDESD; 5001-801 Vila Real, Portugal; diogoamcoutinho@gmail.com (D.C.); ajaime@utad.pt (J.S.); 3 Castelo Branco Football Association, Research Department, 6000-050 Castelo Branco, Portugal; 4 Departamento de Desporto e Saúde, Escola de Ciências e Tecnologia, Universidade de Évora, 7000-645 Évora, Portugal; bgoncalves@uevora.pt; 5 Comprehensive Health Research Center (CHRC), Universidade de Évora, 7000-645 Évora, Portugal; 6 Portuguese Football Federation, Portugal Football School, 1495-433 Oeiras, Portugal; 7 Sports Science School of Rio Maior—Polytechnic Institute of Santarém, 2040-413 Rio Maior, Portugal; rafaeloliveira@esdrm.ipsantarem.pt; 8 Life Quality Research Centre, Polytechnic Institute of Santarém, 2040-413 Rio Maior, Portugal

**Keywords:** exercise duration, regulation of effort, tactical behavior, team behavior, pacing strategy

## Abstract

The study aimed to identify how the manipulation of knowledge regarding a training task duration constrains the pacing and tactical behaviour of soccer players when playing small-sided games (SSG). Twenty professional and experienced soccer players participated in a cross-sectional field study using three conditions: not informed on the duration of the SSG, which ended after 20 min (Unknown Condition); briefed about playing the SSG for 10 min, but after they completed the 10-min game, they were requested to complete another 10 min (Partial Condition) and informed before that they would play for 20 min (Known Condition). A global positioning system was used to measure the total distance covered and distances of different exercise training zones (walking to sprinting) and to access the dynamic players positioning through the distance from each player to all the teammates and opponents. Additionally, approximate entropy was measured to identify the regularity pattern of each gathered individual variable. The results indicate that the first 10 min of each scenario presented a higher physical impact independently of the initial information. During this time, the tactical behaviour also revealed higher variability. An increase in the distance of the teammates during the second period of 10-min for the Known scenario was also found, which may result from a lower pacing strategy. This study showed that the prior knowledge of the task duration led to different physical and tactical behaviours of the players. Furthermore, the relationship between the physical impact and the regularity of team game patterns should be well analysed by the coach, because the physical impact may be harmful to the development of the collective organization of the team.

## 1. Introduction

In team sports, like soccer, the analysis of pacing and tactical behaviour is a key issue to understand players’ and team’s performances [1,2,3,4,5,6]. Due to the need to constantly forge couplings with teammates and break couplings with opponents in space and time to ensure numerical or spatial-temporal advantages [7], the soccer game is characterized by players’ constant positional adjustments at different speeds that create a specific intermittent behaviour with certain pacing [1,2,8]. Notably, the phenomenon of pacing is regarded as the resource of the spreading of energy for the optimization of performance related to match or exercise running requirements. In the past, the variations on running performances in soccer were sustained by the classical theory of fatigue [1,2,9]. However, recent studies have argued that the ability of a player to efficiently manage their effort during competition or training sessions is rather affected by the constant adaptation of players to the changes on the information that sustain their individual and collective behaviour according to their general capabilities, to ensure functional performances [10]. In this process, individual regulation is a neuropsychological process in which afferent sensations contribute to the regulation of exertion [11]. In fact, it is a holistic process in which neuropsychological interactions affect the decisions made about the perception of exercising [3].

The duration of an exercise affects a player’s pacing, which explains the reason why it has been considered as a regulator of performance [12]. For example, when the exercise duration is short, players tend to adopt a more aggressive pacing pattern [1,13]. Additionally, according to some studies [1], while the players who perform the whole match (90 min) start the game integrating a slow-based pacing strategy, followed by a gradual decrease in the intensity of running to maximize the physical performance over the match, in turn, the players who play a part of the match engage in a high intensive pacing strategy as they strive to give all. In addition, deception-based studies (participants believe they are exercising for a given time period but are asked to continue exercising longer towards the completion of this time) show that when the deception is known, their perception of the exertion rate increase, triggering a tremendous decline in the pacing speed. Moreover, some studies reported that players hold some physiological reserves when they are not certain of the duration of the exercises [11,14,15]. Consequently, decisions that pertain to the reduction, increase, or maintenance of certain performances are determined by the momentary and potential “payoff”.

In team sports and particularly in soccer, however, the pacing phenomenon should be viewed as a situational process with implications on players’ perception about individual and collective possibilities for action [16] and consequently on variations on tactical behavior between players and teams. That is, changes in players’ game pace will constrain the spatial–temporal relations between teammates in relation to opponents leading to the emergence of new patterns of play [8,17]. Although pacing can be influenced by the duration and knowledge of the duration that a player has been exposed to exercise, the capability of players to adjust their tactical actions to the game spatial–temporal relations forged with team-mates and opponents at different game pacing seems to be crucial for a successful performance, especially in exercises where fast decision-making and actions are required [18]. Previous research on the analysis of teams’ tactical behaviour revealed that changes from slow to normal or fast game pacing clearly constrain the stability of spatial–temporal relations between teammates during performance [8]. Accordingly, and considering that the prior knowledge of the task duration affects the players’ game pace, it may be expected that these changes in the players’ game pace may also impact the interpersonal coordination between players. However, to our knowledge, there is no information about how such manipulation constrains the tactical behaviour of the players and teams. As such, a better understanding of the players’ movement behaviour would emerge if the complementary relationship between the pacing patterns and the tactical behaviours of players was considered. Therefore, the aim of this study was to investigate how the manipulation of knowledge regarding exercise duration constrains tactical behaviour and pacing behaviour of teams. We hypothesized that the use of prior information regarding the task duration constrains the pacing behaviour and the dynamics of tactical behaviour over the time of the exercise. It might also be expected that higher pacing behaviour causes higher irregularity on the tactical behaviour of teams.

## 2. Materials and Methods

### 2.1. Participants

The study sought for twenty professional soccer players (age 22.3 years ± 2.1; body height 1.82 m ± 0.06; body mass 73 kg ± 5.8) with 10.3 years ± 3.4 of experience, playing in the second division of the Portuguese National Competition. The players were engaged in five training sessions each week totaling 450 min of training, with an additional official game over the weekend. Participants were informed of the study design and its requirements, as well as the possible benefits and risks, and gave their consent prior to the start of the study in accordance with the principles of the Declaration of Helsinki for the study of humans. The study was approved by the local ethical committee (University of Beira Interior, No 275 319 700).

### 2.2. Design

A cross-sectional field study was used and players were tested over four training sessions. In the first session, they were familiarized with the procedures, equipment, and the game. One week after, the players engaged in three randomized small-sided games (SSG) separated by seven days. In the first game scenario, players were not informed on the duration of the SSG, which ended after 20 min (Unknown Condition). In the following game scenario, players were briefed about playing the SSG for 10 min. However, soon after they completed the 10-min game, the participants were requested to complete another 10 min making the total exercise duration of 20 min (Partial Condition). In the third game scenario, players were informed before the game that they would play for 20 min (Known Condition). To control for circadian variations on the measured variables, all games were performed at the same time of the day (from to 17:00 p.m. until 19:00 p.m.), and during these sessions, the average temperature recorded was 18 °C.

### 2.3. Experimental Procedures

During each session, and after a standard 15-min warm-up, an SSG scenario was played. The participants were grouped into 4 balanced teams of 5 players (without goalkeepers) based on the coach perception of the players’ physical, technical, and tactical skills. The same team structure was kept during all the data collection period. The experimental training sessions were performed exactly in the same moment of the microcycle (e.g., Match + 3) during competitive season and the players were always fully rested. Teams’ constitutions and respective opponents were maintained during all conditions. The aim of the game was to outscore the opponents. The small-sided game was played in a standardized playing pitch (20 m wide × 40 m long). Multiple balls were positioned around the field so that in the case of a ball leaving the field, another was quickly introduced to ensure the game continued. The time of the recovery period between the games was always the same (90 s), and no coach feedback or encouragement was allowed.

A total of 20 GPS units recorded players’ tracking displacements based on a sampling frequency of 5 Hz (SPI-Pro X II, GPS ports, Canberra, ACT, and Australia) [19]. The dynamic players’ positioning was gathered using a non-differential 5Hz GPS system and used to compute teams’ tactical behavior. The variables calculated were the distance from each player to all teammates (Distance Player–Teammate) and opponents (Distance Player–Opponent). This calculations comprised all dyads during the games (i.e., the interpersonal distance between each pair of players, both with teammates and opponents) and were calculated by computing the norm between the vectors using the following equation (Equation (1)):

(1) $$D\left(a_{x_{\left(t\right)},y_{\left(t\right)}},b_{x_{\left(t\right)},y_{\left(t\right)}}\right)=\sqrt{{\left(a_{x_{\left(t\right)}}-b_{x_{\left(t\right)}}\right)}^{2}+{\left(a_{y_{\left(t\right)}}-b_{y_{\left(t\right)}}\right)}^{2}}$$

where *D* is the distance, *a* is the player, *x* and *y* are the coordinates, and *t* is the time, and *b* is the teammate or opponent. Moreover, distances were also analyzed according to the amount of variability expressed by the coefficient of variation and according to the structure of the variability using Approximate entropy (ApEn) [20,21].

Approximate entropy (ApEn) was measured to identify the regularity pattern of each gathered individual variable. Input values for computations were 2 to the vector length (m) and 0.2 standard deviations to the tolerance factor (r) [22]. The outcome range between 0 and 2 (arbitrary units) and lower values represented more repeatable, regular, predictable, and less chaotic sequences of data points [22]. From a processing approach, ApEn expresses the probability of the configuration of one segment of data in a time series will allow the prediction of the configuration of another segment of the time series a certain distance apart [23]. The length of the time series used to process the ApEn of each variable was 3000 data sets (10 min of task duration × 60 s × 5Hz).

The external workload was assessed through total distance covered by players and distance covered at different movement speed categories: 0.0–3.5 km/h (walking); 3.6–14.3 km/h (jogging); 14.4–19.7 km/h (running); and >19.8 km/h (sprinting) [24].

### 2.4. Statistical Analysis

A descriptive analysis was performed using mean and standard deviations. Differences in means, i.e., 1st vs. 2nd 10 min bouts for each condition, were expressed in percept units with 90 percent confidence limits (CL). The smallest worthwhile differences were estimated from the standardized units multiplied by 0.2. Uncertainty in the true differences of the scenarios was assessed using non-clinical magnitude-based decisions [25,26,27]. Additionally, the comparisons were assessed via standardized mean differences and respective 90 percent confidence intervals [28]. Thresholds for effect size statistics were 0.2, trivial; 0.6, small; 1.2, moderate; 2.0, large; and 2.0, very large [25,26,27,28].

## 3. Results

### 3.1. Unknown Scenario

**Figure 1:**
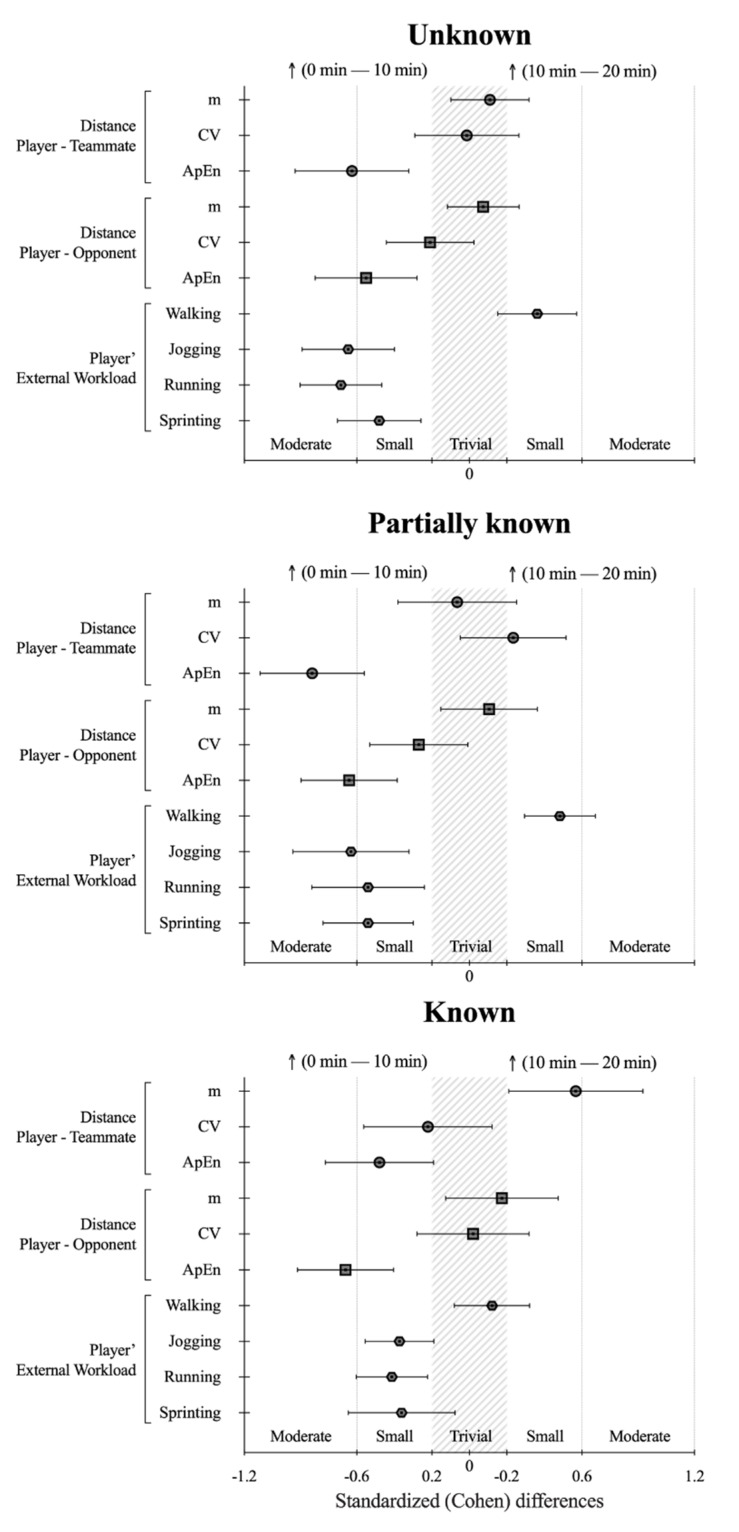
Standardized Cohen’s differences for comparative results of the (0 min–10 min) vs. (10 min–20 min) in the different condition game variables. Error bars indicate uncertainty in true mean changes with 90% confidence intervals. Abbreviations: m = meters; CV = coefficient of variation; ApEn = approximate entropy.

Table 1 and Figure 1 (upper panel), respectively, show the descriptive statistics and standardized differences of the external workload and positioning-derived variables when comparing the (0 min–10 min) with (10 min–20 min) for the Unknown scenario. The absolute Distance Player–Teammate and the respective coefficient of variation revealed unlikely and unclear differences, respectively. However, for the same variable, moderate lower values of the ApEn were observed during the (10 min–20 min) (i.e., players were more regular in their positioning within the teammates) in comparison with the (0 min–10 min). The absolute Distance Player–Opponent revealed likely trivial values during the (10 min–20 min) in comparison with the (0 min–10 min). In opposition, there was a small higher coefficient of variation and ApEn in the (10 min–20 min) in comparison with the (0 min–10 min). These results were complemented by the small higher distance covered while walking (means variation, % ± 90 confidence limits: 10.4% ± 5%), moderate lower while jogging (−10.6% ± 4.8%), and small lower running and sprinting during (0 min–10 min) than in (10 min–20 min).

### 3.2. Partially Known Scenario

Table 2 and Figure 1 (middle panel), respectively, show the descriptive statistics and standardized differences of the external workload and positioning-derived variables when comparing the (0 min–10 min) with (10 min–20 min) for the Partially known scenario. The absolute Distance Players–Teammate covered was unclear during both periods, the (0 min–10 min) presented small higher values in the coefficient of variation within moderate-higher values in the ApEn. The absolute distance players–opponents covered were observed as trivial between both periods with small lower values in the coefficient of variation and moderate lower approximate entropy in the (10 min–20 min). For the same trend in absolute distances in both periods, a different external workload was demanded. The small higher distance covered while walking was (17.0% ± 9.8%), moderate lower while jogging (−10.6% ± 4.8%), and small lower in both running and sprinting (−22.5% ± 14.4% and −33% ± 32%, respectively) were found during the (10 min–20 min).

### 3.3. Known Scenario

The descriptive statistics and standardized differences for considered variables in the Known scenario are shown in both Table 3 and Figure 1 (lower panel), respectively. While small to moderate lower absolute distance players–teammates were found for the (10 min–20 min), the coefficient of variation and approximate entropy presented opposite trends, by showing small lower values compared to the (0 min–10 min). The Known condition was the scenario that revealed fewer effects in the distance players–opponents’ variables, whereas moderate lower approximate entropy was only found during the (10 min–20 min). Within the increase in the players–teammates distances in the (10 min–20 min), a small lower distance covered while jogging (−7.4% ± 3.0%) and running (−17.7% ± 12.4%) was found. In addition, a small higher distance covered while walking (10.4% ± 5.0%) was identified during this period.

## 4. Discussion

The study aimed to understand how the manipulation of knowledge regarding task duration constrains the pacing and tactical behaviour of soccer players when playing small-sided games. From the results obtained, it was evident that the first 10 min of each scenario presented a higher physical impact in comparison with the second 10 min for all the conditions considered. During this time, the tactical behaviour also revealed higher irregularity, promoting high variability in the collective team organization. Additionally, an increase in the distance of the teammates during the second period of 10 min (10 min–20 min) for the Known scenario was also found, which may result from a lower pacing strategy.

The current study identified a tendency: that most of the external load variables revealed higher values during the first 10 min exercise duration of any scenario. This trend is supported by Gabbett, Walker and Walker [29], Highton, Mullen and Twist [30] and Ferraz et al. [1] where most of the physical variables showed a tendency to be higher in the first few minutes of the exercise. Indeed, similar results were also verified in sprinting analysis [31]. The findings from this study also corroborate that the player’s knowledge of the exercise’ duration influences the players’ pacing pattern [31]. Overall, it seems that a self-regulation strategy of the regulation of effort was developed based on prior knowledge of the task.

According to the results, it can also be suggested that players seem to adopt a “slow positive” pacing profile characterised by a gradual decline in the total and high-intensity running between the initial and the second exercise period [1,2,32]. Furthermore, as pointed out in some similar studies, the differences between the first 10 min and the last 10 min tend to decrease when the knowledge of the task duration increases. This means that most of the variables have a tendency to be higher in the initial period of the task but there is a better balance in the regulation of effort when the knowledge is greater [1,2]. Likewise, the results may confirm the possibility of the changes in the pacing patterns of the players due to the effect of the knowledge of the task duration that leads to the possibility of the non-linearity of the fatigue effect already pointed out in some studies [33,34]. This means that the decision to reduce or increase the efferent neural drive manifested in a decrease or increase in physical performance is based on the degree of uncertainty or certainty about the task duration and the degree of potential homeostasis disruption perceived in each condition, in connection with afferent feedback and feed forward-systems [35]. Indeed, some studies also revealed that responses to exercise without knowledge of duration may reflect a subconscious improvement in exercise economy to conserve energy, owing to the unknown duration of the exercise bout [12,36,37].

The players’ movement behaviour on the pitch also results from their interaction with the surrounding information and with the key task constraints, such as the prior knowledge of task duration analysed [2,38,39]. Accordingly, a better understanding of the players’ movement behaviour may be achieved when it is considered as the complementary relation between tactical behaviour and the players’ physical performance [17,40,41] Generally, the results of this study revealed higher irregularity in the patterns of the distance of the teammates and in the distance of the opponents during the first 10 min (0 min–10 min) compared to the second period of 10 min (10 min–20 min) in all game conditions. Additionally, a higher distance covered in high-intensity during the first 10 min (0–10 min) was observed. Thus, it is possible that the higher game pace may have affected the stability of players’ interpersonal distances. Similar results were found by Sampaio et al. [8], who found higher regular distances during SSG conditions performed at slower game paces, suggesting that games performed at faster paces may impair the players’ tactical behaviour. That is, considering that players’ actions are dependent on their ability to use the relevant information from the environment [39,42,43,44], fast pace games seem to require a faster ability to identify this information, which may affect the players’ positional behaviour [8]. In fact, it has been shown that players reveal high levels of team coordination at lower speeds [45]. Therefore, these results must be seen not only at the individual level, but also at the team level, since it also requires that all teammates are able to understand and use the same information to achieve functional, collective movement behaviours [46,47].

Interestingly, while no differences in the absolute distance of the teammates were found when comparing the first 10 min (0 min–10 min) to the second period of 10 min (10 min–20 min) for the Unknown and Partially known scenarios, in turn, the Known condition showed an increase in the distance to the teammates during the second period (10 min–20 min). This can be speculated on that the knowledge about the time of practice afford a spread of players’ distribution on the field and an increase in positional behavior of players, supported by the decrease in the game pace. That is, the increase in the distance of the teammates may be related to the more conscious change in players pacing strategy. The prior knowledge of longer task duration may create a higher effort regulation capability, leading the players to increase the exercise economy by improving positional relationships [2]. A similar increase in positional behaviours occurs when teams faced congested fixtures in comparison with the regular play of one game per week, as a strategy to maintain a high level of performance even with lower physical availability to play [40]. A possible explanation could be related to the decrease of players’ focus of attention on adjusting their positioning based on the distance of the teammates in order to focus on more relevant information, such as the ball location and the space available to play [48,49]. Furthermore, increasing the distance of the teammates, in this case, may result in a functional strategy to cover a great pitch space, improving physical exercise economy. In this regard, coaches must acknowledge that, during longer training tasks, mainly if there is prior knowledge of the task duration, it seems that players adopt a pacing strategy to be able to cope with the task demands until the end. This might lead to the different exploration of tactical possibilities for action, and consequently, different couplings with teammates and opponents in space and time to ensure numerical or spatial-temporal advantages [7].

This study highlights the importance of the knowledge of the task duration on the pacing and tactical behaviours of soccer players. The relationship between the physical impact and the regularity of team game patterns should be well analysed by the coach. If, on one hand, the conditional abilities for a good physical condition for the player is required, on the other hand, the physical impact may be harmful to the development of the collective organization of the team. For example, short periods of exercise (e.g., blocks of 10 min), resulted in higher physical stimulus, independent of initial knowledge of the task duration and, additionally, in higher variability in the patterns of the distance of the teammates and in the distance of the opponents from a tactical perspective. Furthermore, while the Unknown and Partially known scenarios revealed similar movement behaviours, it seems that knowing the task duration led to an increase in the players’ distance to the teammates during the last part of longer exercise duration (for example, the second period of 10 min (10 min–20 min)) due to lower pacing patterns. Overall, the information on the prior task knowledge of the task duration is an important variable that coaches should consider since it affects the physical performance of players, and consequently, the players’ movement behaviour. Therefore, coaches should be careful not only in defining the duration of the training tasks, but also in deciding if the players should be aware of its duration, as this led to different physical outcomes and different exploration of players’ and teams’ possibilities for action.

## 5. Conclusions

Overall, the results from this study show that the prior knowledge of the task duration leads to different physical and tactical behaviours of the players. For instance, a lower regularity in the players’ distance to the teammates and opponents was found during the first 10 min (0–10 min) compared to the second period of 10-min (10 min–20 min) for all game scenarios, which seems to be related with the higher physical impact (higher game pace) found during the first period of 10 min. Additionally, an increase in the distance to the teammates during the second period of 10 min (10 min–20 min) was also found for the Known scenario, which may result from a lower pacing strategy. That is, the increase in the distance of the teammates may be related to the more conscious change in players pacing strategy. The prior knowledge of longer task duration may create a higher effort regulation capability leading the players to increase the exercise economy by improving positional relationships. In this scenario, players may decrease their focus on the distance of the teammates, to be able to focus on more relevant information, such as the ball location and the space available to play, helping them to perform functional movement behaviours. These results provide important information regarding the prior knowledge of the task duration, which should be considered by coaches during training practices.

## Figures and Tables

**Table 1:** Descriptive statistics when comparing (0 min–10 min) vs. (10 min–20 min) for the Unknown condition game variables.

Variables	Unknown						
	(0 min–10 min)	(10 min–20 min)	Change in Mean % ± 90% CL	Chances, %			Practical Inferences
	Mean ± SD	Mean ± SD		↓	Trivial	↑	
**Distance Player—Teammate**							
m	12.2 ± 1.6	12.3 ± 1.5	1.3 ± 2.6	1	75	24	unlikely
CV	0.45 ± 0.06	0.45 ± 0.05	−0.2 ± 3.3	14	76	10	unclear
ApEn	0.21 ± 0.03	0.19 ± 0.03	−10.0 ± 4.5	99	1	0	very likely ↓
**Distance Player—Opponent**							
m	12.0 ± 1.9	12.2 ± 2.2	1.2 ± 3.2	1	85	14	likely trivial
CV	0.49 ± 0.06	0.47 ± 0.07	−3.1 ± 3.4	54	46	0	possibly ↓
ApEn	0.20 ± 0.03	0.18 ± 0.03	−9.1 ± 4.6	97	3	0	very likely ↓
**Players’ External Workloads**							
Walking	100.3 ± 30.1	111.7 ± 37.2	10.4 ± 5	0	10	90	likely ↑
Jogging	859.1 ± 135.6	758 ± 148.9	−12.3 ± 3.4	100	0	0	most likely ↓
Running	115.4 ± 49	92.1 ± 50.4	−27.4 ± 14	95	5	0	very likely ↓
Sprinting	17.9 ± 16.9	10.1 ± 9.1	−36.0 ± 30.4	85	13	2	likely ↓

Abbreviations: m = meters; CV = coefficient of variation; ApEn = approximate entropy; CL = Confidence limits; ↓ = lower; ↑ = higher.

**Table 2:** Descriptive statistics when comparing (0 min–10 min) vs. (10 min–20 min) for the Partially known condition game variables.

Variables	Partially Known						
	(0 min–10 min)	(10 min–20 min)	Change in Mean % ± 90% CL	Chances, %			Practical Inferences
	Mean ± SD	Mean ± SD		↓	Trivial	↑	
**Distance Player–Teammate**							
m	12.4 ± 1.2	12.4 ± 1.5	−0.7 ± 3.4	24	67	9	unclear
CV	0.45 ± 0.04	0.46 ± 0.05	2.3 ± 2.8	1	41	58	possibly ↑
ApEn	0.21 ± 0.03	0.18 ± 0.03	−11.8 ± 3.6	100	0	0	most likely ↓
**Distance Player–Opponent**							
m	12.2 ± 1.8	12.4 ± 2.1	1.7 ± 4.1	3	70	27	possibly ↑
CV	0.48 ± 0.06	0.46 ± 0.06	−3.6 ± 3.5	68	32	0	possibly ↓
ApEn	0.2 ± 0.03	0.18 ± 0.03	−9.3 ± 3.7	100	0	0	most likely ↓
**Players’ External Workloads**							
Walking	93.1 ± 28.5	110.6 ± 40.1	17 ± 9.8	0	4	96	very likely ↑
Jogging	845.8 ± 107.9	763.5 ± 141.3	−10.6 ± 4.8	99	1	0	very likely ↓
Running	120.4 ± 46.8	97.5 ± 49.4	−22.5 ± 14.4	91	9	0	likely ↓
Sprinting	24.8 ± 21.2	16.8 ± 13.4	−33 ± 32	80	18	2	likely ↓

Abbreviations: m = meters; CV = coefficient of variation; ApEn = approximate entropy; CL = Confidence limits; ↓ = lower; ↑ = higher.

**Table 3:** Descriptive statistics when comparing (0 min–10 min) vs. (10 min–20 min) for the Known condition game variables.

Variables	Known						
	(0 min–10 min)	(10 min–20 min)	Change in Mean % ± 90% CL	Chances, %			Practical Inferences
	Mean ± SD	Mean ± SD		↓	Trivial	↑	
**Distance Player–Teammate**							
m	12.2 ± 1.2	12.9 ± 1.3	5.9 ± 3.9	0	4	96	very likely ↑
CV	0.45 ± 0.05	0.44 ± 0.04	−2.1 ± 3.1	56	42	2	possibly ↓
ApEn	0.2 ± 0.03	0.18 ± 0.03	−7.3 ± 4.1	95	5	0	very likely ↓
**Distance Player–Opponent**							
m	12.0 ± 1.9	12.3 ± 1.9	2.6 ± 4.6	2	53	45	possibly ↑
CV	0.48 ± 0.07	0.48 ± 0.06	0.2 ± 4.4	12	73	15	unclear
ApEn	0.20 ± 0.04	0.18 ± 0.02	−9.6 ± 3.7	100	0	0	most likely ↓
**Players’ External Workloads**							
Walking	100.3 ± 30.1	111.7 ± 37.2	10.4 ± 5	0	10	90	likely ↑
Jogging	815.3 ± 126.7	755.1 ± 117.4	−7.4 ± 3	99	1	0	very likely ↓
Running	107.5 ± 37.2	93.7 ± 45.5	−17.7 ± 12.4	87	13	0	likely ↓
Sprinting	22 ± 17.4	20.9 ± 15.3	−10.8 ± 37	40	49	11	unclear

Abbreviations: m = meters; CV = coefficient of variation; ApEn = approximate entropy; CL = Confidence limits; ↓ = lower; ↑ = higher.

## References

[1] Ferraz R., Gonçalves B., Van Den Tillaar R., Jiménez Sáiz S., Sampaio J., Marques M.C. (2017). Effects of knowing the task duration on players’ pacing patterns during soccer small-sided games. J. Sports Sci..

[2] Ferraz R., Gonçalves B., Coutinho D., Marinho D.A., Sampaio J., Marques M.C. (2018). Pacing behaviour of players in team sports: Influence of match status manipulation and task duration knowledge. PLoS ONE.

[3] Foster C., Hector L.L., Welsh R., Schrager M., Green M.A., Snyder A.C. (1995). Effects of specific versus cross-training on running performance. Eur. J. Appl. Physiol. Occup. Physiol..

[4] Haddad M., Chaouachi A., Wong D.P., Castagna C., Hambli M., Hue O., Chamari K. (2013). Influence of fatigue, stress, muscle soreness and sleep on perceived exertion during submaximal effort. Physiol. Behav..

[5] Scott B.R., Lockie R.G., Knight T.J., Clark A.C., Janse de Jonge X.A.K. (2013). A comparison of methods to quantify the in-season training load of professional soccer players. Int. J. Sports Physiol. Perform..

[6] Oliveira R., Brito J., Martins A., Mendes B., Calvete F., Carrico S., Ferraz R., Marques M.C. (2019). In-season training load quantification of one-, two- and three-game week schedules in a top European professional soccer team. Physiol. Behav..

[7] McGarry T., Anderson D.I., Wallace S.A., Hughes M.D., Franks I.M. (2002). Sport competition as a dynamical self-organizing system. J. Sports Sci..

[8] Sampaio J.E., Lago C., Gonçalves B., Maçãs V.M., Leite N. (2014). Effects of pacing, status and unbalance in time motion variables, heart rate and tactical behaviour when playing 5-a-side football small-sided games. J. Sci. Med. Sport.

[9] Vigne G., Gaudino C., Rogowski I., Alloatti G., Hautier C. (2010). Activity profile in elite Italian soccer team. Int. J. Sports Med..

[10] Travassos B., Davids K., Araújo D., Esteves T.P. (2013). Performance analysis in team sports: Advances from an Ecological Dynamics approach. Int. J. Perform. Anal. Sport.

[11] Eston R., Stansfield R., Westoby P., Parfitt G. (2012). Effect of deception and expected exercise duration on psychological and physiological variables during treadmill running and cycling. Psychophysiology.

[12] Edwards A.M., Noakes T.D. (2009). Dehydration: Cause of fatigue or sign of pacing in elite soccer?. Sports Med..

[13] Abade E.A., Goncalves B.V., Leite N.M., Sampaio J.E. (2014). Time-motion and physiological profile of football training sessions performed by under-15, under-17 and under-19 elite Portuguese players. Int. J. Sports Physiol. Perform..

[14] Marcora S.M. (2008). Do we really need a central governor to explain brain regulation of exercise performance?. Eur. J. Appl. Physiol..

[15] Sullivan C., Bilsborough J.C., Cianciosi M., Hocking J., Cordy J., Coutts A.J. (2014). Match score affects activity profile and skill performance in professional Australian Football players. J. Sci. Med. Sport.

[16] Coutinho D., Gonçalves B., Travassos B., Wong D.P., Coutts A.J., Sampaio J.E. (2017). Mental Fatigue and Spatial References Impair Soccer Players’ Physical and Tactical Performances. Front. Psychol..

[17] Goncalves B., Esteves P., Folgado H., Ric A., Torrents C., Sampaio J. (2017). Effects of Pitch Area-Restrictions on Tactical Behavior, Physical, and Physiological Performances in Soccer Large-Sided Games. J. Strength Cond. Res..

[18] Faulkner J., Arnold T., Eston R. (2011). Effect of accurate and inaccurate distance feedback on performance markers and pacing strategies during running. Scand. J. Med. Sci. Sports.

[19] Johnston R.J., Watsford M.L., Pine M.J., Spurrs R.W., Murphy A.J., Pruyn E.C. (2012). The validity and reliability of 5-Hz global positioning system units to measure team sport movement demands. J. Strength Cond. Res..

[20] Low B., Coutinho D., Gonçalves B., Rein R., Memmert D., Sampaio J. (2020). A Systematic Review of Collective Tactical Behaviours in Football Using Positional Data. Sport. Med..

[21] Gonçalves B.V., Figueira B.E., Maçãs V., Sampaio J. (2013). Effect of player position on movement behaviour, physical and physiological performances during an 11-a-side football game. J. Sports Sci..

[22] Yentes J.M., Hunt N., Schmid K.K., Kaipust J.P., McGrath D., Stergiou N. (2013). The appropriate use of approximate entropy and sample entropy with short data sets. Ann. Biomed. Eng..

[23] Harbourne R.T., Stergiou N. (2009). Movement variability and the use of nonlinear tools: Principles to guide physical therapist practice. Phys. Ther..

[24] Folgado H., Duarte R., Fernandes O., Sampaio J. (2014). Competing with lower level opponents decreases intra-team movement synchronization and time-motion demands during pre-season soccer matches. PLoS ONE.

[25] Batterham A.M., Hopkins W.G. (2006). Making meaningful inferences about magnitudes. Int. J. Sports Physiol. Perform..

[26] Hopkins W.G., Marshall S.W., Batterham A.M., Hanin J. (2009). Progressive statistics for studies in sports medicine and exercise science. Med. Sci. Sports Exerc..

[27] Hopkins W.G. (2019). Compatibility Intervals and Magnitude-Based Decisions for Standardized Differences and Changes in Means. Sportscience.

[28] Cumming G. (2012). Understanding the New Statistics: Effect Sizes, Confidence Intervals, and Meta-Analysis.

[29] Gabbett T.J., Walker B., Walker S. (2015). Influence of prior knowledge of exercise duration on pacing strategies during game-based activities. Int. J. Sports Physiol. Perform..

[30] Highton J., Mullen T., Twist C. (2017). Knowledge of Task End-Point Influences Pacing and Performance During Simulated Rugby League Match-Play. Int. J. Sports Physiol. Perform..

[31] Billaut F., Bishop D.J., Schaerz S., Noakes T.D. (2011). Influence of knowledge of sprint number on pacing during repeated-sprint exercise. Med. Sci. Sports Exerc..

[32] Waldron M., Highton J. (2014). Fatigue and pacing in high-intensity intermittent team sport: An update. Sport. Med..

[33] Ferraz R., van den Tillaar R., Marques M.C. (2012). The effect of fatigue on kicking velocity in soccer players. J. Hum. Kinet..

[34] Millet G.Y. (2011). Can neuromuscular fatigue explain running strategies and performance in ultra-marathons? The flush model. Sports Med..

[35] Renfree A., Martin L., Micklewright D., St Clair Gibson A. (2014). Application of decision-making theory to the regulation of muscular work rate during self-paced competitive endurance activity. Sports Med..

[36] Swart J., Lindsay T.R., Lambert M.I., Brown J.C., Noakes T.D. (2012). Perceptual cues in the regulation of exercise performance—Physical sensations of exercise and awareness of effort interact as separate cues. Br. J. Sports Med..

[37] Meeusen R., Watson P., Hasegawa H., Roelands B., Piacentini M.F. (2006). Central fatigue: The serotonin hypothesis and beyond. Sports Med..

[38] Travassos B., Araujo D., Davids K., Vilar L., Esteves P., Correia V. (2012). Informational constraints shape emergent functional behaviors during performance of interceptive actions in team sports. Psychol. Sport Exerc..

[39] Travassos B., Duarte R., Vilar L., Davids K., Araujo D. (2012). Practice task design in team sports: Representativeness enhanced by increasing opportunities for action. J. Sports Sci..

[40] Folgado H., Gonçalves B., Sampaio J. (2018). Positional synchronization affects physical and physiological responses to preseason in professional football (soccer). Res. Sports Med..

[41] Gonçalves B., Coutinho D., Travassos B., Folgado H., Caixinha P., Sampaio J. (2018). Speed synchronization, physical workload and match-to-match performance variation of elite football players. PLoS ONE.

[42] Gonçalves B., Marcelino R., Torres-Ronda L., Torrents C., Sampaio J. (2016). Effects of emphasising opposition and cooperation on collective movement behaviour during football small-sided games. J. Sports Sci..

[43] Ric A., Hristovski R., Gonçalves B., Torres L., Sampaio J., Torrents C. (2016). Timescales for exploratory tactical behaviour in football small-sided games. J. Sports Sci..

[44] Ric A., Torrents C., Gonçalves B., Torres-Ronda L., Sampaio J., Hristovski R. (2017). Dynamics of tactical behaviour in association football when manipulating players’ space of interaction. PLoS ONE.

[45] Coutinho D., Goncalves B., Santos S., Travassos B., Wong D.P., Sampaio J. (2019). Effects of the pitch configuration design on players’ physical performance and movement behaviour during soccer small-sided games. Res. Sports Med..

[46] Passos P., Araújo D., Davids K. (2016). Competitiveness and the Process of Co-adaptation in Team Sport Performance. Front. Psychol..

[47] Reimer T., Park E.S., Hinsz V.B. (2006). Shared and Coordinated Cognition in Competitive and Dynamic Task Environments: An Information-Processing Perspective for Team Sports. Int. J. Sport Exerc. Psychol..

[48] Folgado H., Bravo J., Pereira P., Sampaio J. (2019). Towards the use of multidimensional performance indicators in football small-sided games: The effects of pitch orientation. J. Sports Sci..

[49] Headrick J., Davids K., Renshaw I., Araújo D., Passos P., Fernandes O. (2012). Proximity-to-goal as a constraint on patterns of behaviour in attacker–defender dyads in team games. J. Sports Sci..

